# Do Websites Provide What Applicants Need? Plastic Surgery Residency Program Websites Versus Applicant Self-reported Needs

**DOI:** 10.1097/GOX.0000000000001900

**Published:** 2018-10-02

**Authors:** Vivi W. Chen, Don Hoang, Warren Garner

**Affiliations:** From the Department of Plastic and Reconstructive Surgery, Keck School of Medicine, University of Southern California; Los Angeles, Calif.

## Abstract

**Background:**

Plastic surgery residency program websites (PSRWs) remain a major resource for applicants to one of the most competitive specialties in medicine, yet it is unclear how the website content aligns with information applicants need.

**Objective:**

To evaluate PSRWs and whether they are meeting applicant needs.

**Methods:**

An anonymous online survey of 2016 plastic and reconstructive surgery applicants evaluated the use of PSRWs and information desired. Two researchers then independently analyzed all 93 PSRWs in the United States to determine whether the websites met the needs of applicants based on a quantitative scoring system.

**Results:**

Out of 369 total applicants to plastic surgery, the survey was sent to the 189 applicants to University of Southern California, and 87 responded (46% response rate). Ninety-eight percentage of respondents used websites during the application process. For 31%, websites served as the only source of information. Websites were moderately (60%) and only slightly useful (32%). For 22% and 34%, the quality of a website influenced their decision to apply or interview, respectively. Out of the 22 common components between the applicant survey and scoring assessment of websites, there was a significant difference in 17 components (77%) between those rated as “important” by applicants and availability of the corresponding component on websites (*P* < 0.05). Average (SD) score among websites was 18.7 (5.3) out of 49 maximum points (36.9%). Over 30% of websites displayed broken links or missing or outdated information. Only 19.6% displayed a link to provide website feedback.

**Conclusions:**

PSRWs commonly include pertinent information that is useful; however, most are lacking components valued by applicants. These findings can help guide the design, content, and improvement of PSRWs to be quality resources for applicants.

## Abstract 2

Supplemental Digital Content is available in the text.

## INTRODUCTION

*The New York Times* has praised digital technology for “letting in new voices, creating new formats for exploration, and allowing fans and other creators to participate in a glorious remixing of the work.”^1^ This underscores the broad societal implications of digital technology. Within medical education, the internet serves as a major portal through which medical students gain more insight into different residency programs. Medical schools have created websites providing students with information on possible specialty choices, residency preparation, and career assessment and planning.^2^ A previous study demonstrated that almost 80% of applicants believe that the online presence of a residency program influenced their decision to apply to that program.^3^ Hashmi et al.^3^ and Silvestre et al.^4^ evaluated various components of plastic surgery residency program websites (PSRWs) to determine website quality, and concluded that websites are lacking in content and accessibility. But what would applicants themselves like to see on websites, and, are websites adequately fulfilling this need? Ultimately, there is a paucity of evidence to guide plastic surgery departments in designing quality websites that provide information in line with what applicants desire in their application and program selection process.

The objectives of this study were to (1) use a web survey to investigate information that plastic and reconstructive surgery residency applicants desire to see on PSRWs; and (2) review all PSRWs in the United States to determine whether websites meet the information needs of those applicants.

## METHODS

### Self-reported Needs of Plastic Surgery Residency Program Applicants

Upon USC institutional review board exemption from human subjects research under 45 CFR 46.101(b)2, a request to participate in a web-based survey (SquareSpace, New York, N.Y.) was e-mailed to all 189 plastic and reconstructive surgery residency program applicants to USC for the 2015 to 2016 application cycle (February 23, 2016). Name identifiers were removed, and completed entries were automatically linked to an Excel sheet. Duplicate submissions were manually filtered out. The survey was constructed based on a combination of elements similar to those utilized in previous literature.^3,4^ It included 15 questions, consisting of applicants’ use of residency program websites in their research on programs, any impact on decision-making in applications and interviews, and components of a websites that applicants believed to be most important. Answers incorporated Likert scales, multiple choice, yes-no, or open-ended systems (**see appendix, Supplemental Digital Content 1**, which displays a list and format of survey questions for applicants, http://links.lww.com/PRSGO/A865).

### Measuring PSRW Information Quality of US Plastic Surgery Residency Programs

A scoring system was created to assess the quality of PSRWs in meeting the information needs of plastic surgery residency program applicants (Table 1). Criteria were selected based on previously published literature from various medical specialties, on contents of residency program websites that were important to applicants.^3–7^

**Table 1.**
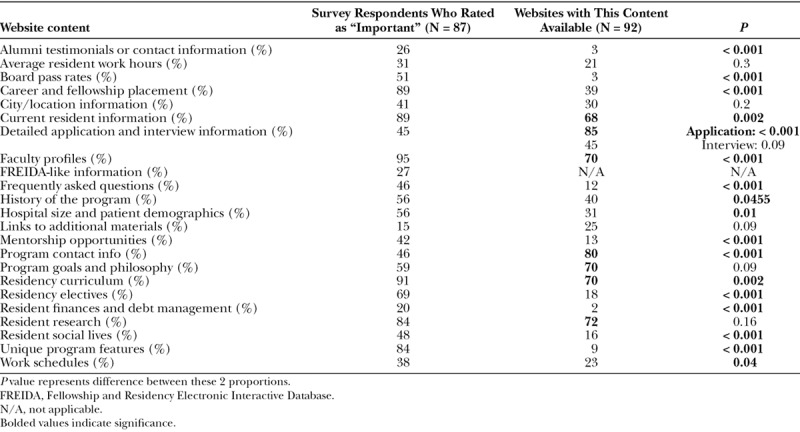
Information on PSRWs that Plastic Surgery Applicants Value Versus Actual Availability on Websites

The American Medical Association Fellowship and Residency Electronic Interactive Database was accessed for a listing of plastic and reconstructive surgery residency programs in February 2016. All included websites were each searched by the authors (D.H. and V.W.C.) for the presence or absence of criteria listed in Table 1, with the addition of components such as broken links, nonfunctional pages, donation links, and website feedback links. Forty-nine components were assessed, and a website was given a score of 1 if the information was present and a score of 0 if the information was unavailable or inaccessible. Information was only considered to be addressed on program sites if it was directly available—links to outside pages were not evaluated.

### Statistical Analysis

Data analysis was largely descriptive; counts and percentages were used for all categorical variable outcomes. If necessary for comparison, responses to Likert scale questions were converted to binary outcomes by grouping responses like “agree” and “strongly agree” together. A value of *P* < 0.05 was considered statistically significant.

## RESULTS

### USC Plastic Surgery Residency Program Applicant Needs

#### Survey Response

Out of 369 total applicants to plastic surgery in the nation,^8^ the survey was sent to the 189 applicants to USC, and 87 responded (46% response rate).

#### Survey Results

**Fig. 1.**
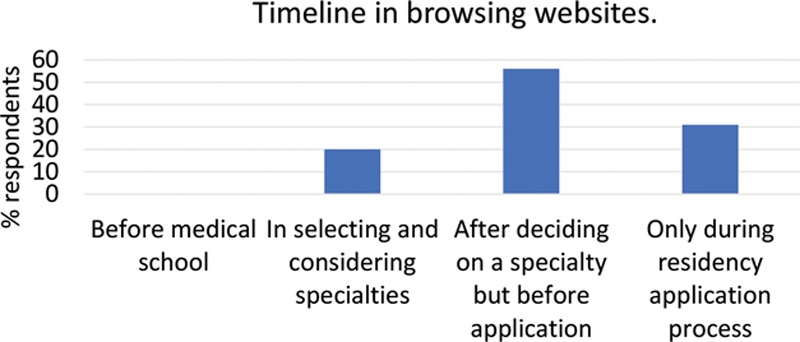
Time during career in which applicants reported using PSRWs.

**Fig. 2.**
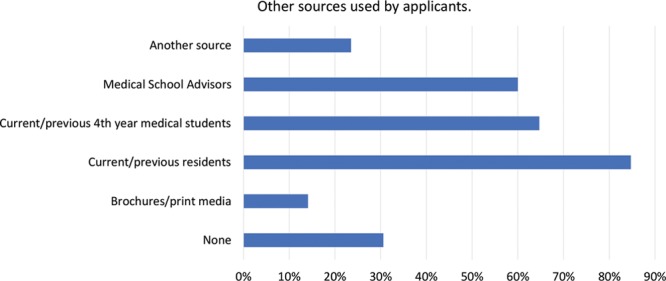
Alternative sources of information used by applicants in researching plastic and reconstructive surgery programs.

**Fig. 3.**
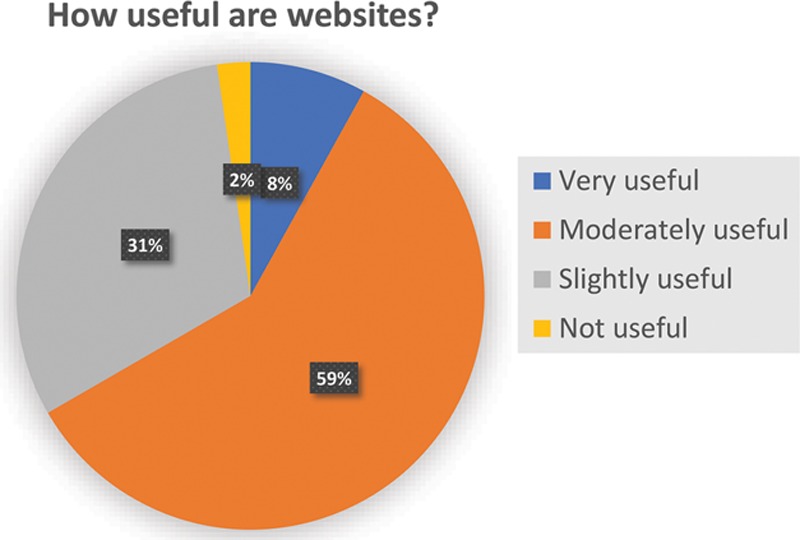
Perceived usefulness of PSRWs by applicants to plastic and reconstructive surgery.

Almost all respondents (98%) used PSRWs during the application process. Most applicants (59%) visited websites after deciding on their specialty but before the application process (Fig. 1). In addition to websites, other resources utilized are detailed in Figure 2. Few applicants found the websites “very useful” (8%; Fig. 3). Over a third of students reported that the quality of the websites influenced their decision to interview (34%), and some students reported that the quality of websites influenced their decision to apply (22%). Of note, many applicants (41%) reported a discrepancy between the website and the interview encounter of the respective program.

Applicants most valued information on: faculty (95%), residency curriculum (91%), current residents (89%), career and fellowship (89%), and resident research (84%; Table 1). Design aspects that applicants most valued included video content (95%), consistent page layouts (87%), aesthetic quality (71%), and programmatic content (63%; Table 2).

**Table 2.**
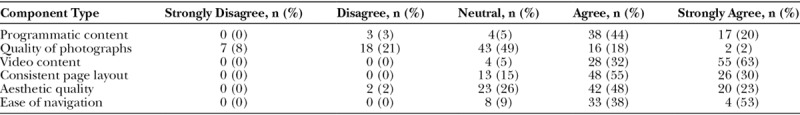
Design and Interactive Components that Applicants Value on PSRWs

### PSRW Information Quality

Of the 93 residency programs, 92 programs had websites available (98.9%). Average score (SD) among all 92 websites was 18.7 (5.3) out of 49 points (36.9%).

Among content categories that applicants rated as important (Table 1), 70% of websites included faculty information, 70% included residency curriculum, 68% included resident information, 39% included career and fellowship information, and 72% included resident research. Out of the 5 website content categories stated above as most valued by applicants, only 1 website included information in all 5. Out of 22 common components between the applicant survey and scoring assessment of websites, there was a significant difference in 17 components (77%) between those rated as “important” by applicants and availability of the corresponding component on websites (*P* < 0.05; Table 1).

Few websites displayed some form of video content (9.8%), significantly different from the number of applicants who found video content important (*P* < 0.001). Over 30% of websites displayed broken links or missing or outdated information. Few websites displayed a link to provide website feedback (19.6%), whereas most websites (62.0%) displayed links to donate to the institution or affiliated hospital.

## DISCUSSION

With the continued impact of technological advances on medical education,^9^ the behaviors and preferences of students are evolving as well. For example, applicants now reportedly find multimedia applications useful for learning about intangible aspects of a residency program that can help them determine fit and make an informed decision about a potential program, such as “how happy people are.”^10^

Of the residency applicants surveyed, almost all utilize websites in their research of residency programs. Correspondingly, almost all plastic surgery residency programs provide a website for their program. However, our study found that most applicants did not find these websites to be optimally useful, and for many applicants, these websites influence their decision to interview. This finding is important considering the competitive nature of applying to this specialty, in which applicants are often recommended to diversify the types of programs at which they interview.^10^ Of note, an important factor of the interview process is that it is resource intensive^11^ for both the residency program and the applicant. Although this does not necessarily imply that applicants prioritize website experience over their training, it demonstrates that websites provide a virtual window into a program and serve as an important preliminary factor in many applicants’ decisions to interview. It is also important to note that the website also does not necessarily take precedence over other factors in deciding where to interview, such as mentor suggestions, geography, or program fit, but it may serve as an initial access for applicants with no preliminary knowledge of a particular program. Interestingly, we found that 41% of applicants reported a discrepancy between the website and their interview experience. It is unclear whether this is due to overall aesthetic appeal of the website or simply inaccurate information. While applicants to plastic surgery traditionally apply broadly, in 2016 there was a decrease in applications per applicant, at 33.4 down from 50.9 the previous year.^8^ Applications per applicant in 2017 remained decreased at 35.5. It is therefore important to consider that plastic surgery applicant trends continue to change.

Furthermore, we found incongruity between applicant needs and website content. Based on the survey, applicants most valued academic and career characteristics, such as career and fellowship placement, faculty profiles, and residency research and curriculum. Most websites adequately provided faculty profiles, curriculum, and resident profiles, but our data identify unique program features, career and fellowship placement, and residency electives as major areas for improvement. Additionally, some applicants valued categories that almost no websites included (< 5%), such as alumni information, board pass rates, and resident finances and debt management. This represents a unique opportunity for websites to stand out if they choose to provide this information. For example, information on finance and debt management, while rarely found on websites, could potentially provide residents with resources for budgeting, estate planning, investment strategies, and retirement planning—all elements that can offer significant long-term benefits. Indeed, when surveyed, residents and fellows demonstrate low financial literacy and investment-risk tolerance, high debt, and deficits in financial preparedness.^12^ Overall, we found misalignment between websites and applicants’ surveyed needs. It is unclear whether this misalignment is because programs are unaware of what applicants value when visiting websites or because website design represent lower priority within a program’s agenda. However, without adequately fitting applicant needs, websites fail to serve their fundamental purpose—that is, as efficient communication tools for visitors.

Another major discrepancy lies in website design and interaction. Applicants value aspects such as video content, page layout, and aesthetic quality, but only a minority of websites provided video content and many websites exhibited functional issues such as broken links, missing or outdated information, or a nonfunctioning page. The use of more interactive and collaborative content continues to increase^13–15^ and websites are progressing from simple text-based applications. Many medical journals, from *The New England Journal of Medicine* to *Journal of American Medical Association*, now share updates on Facebook, postinformational videos on YouTube, and tweet new and free content on Twitter. Therefore, in addition to providing text-based content, programs should consider including more interactive interface on their websites, such as utilizing videos to uniquely present information.

Our study has its limitations. First, our study surveyed 189 applicants to USC out of a total of 369 applicants to plastic and reconstructive surgery overall.^8^ This is similar to the reported average applications per program, which was reported to be 178.5 in 2016. Out of those who responded, it is possible that there is a bias toward those who utilize more technology. It is also likely that there is a geographical bias within this sample, which does not necessarily represent opinions of the entire application pool. Second, while concrete factors such as broken links and interactive content may provide some insight into the aesthetic component of a website, an appropriate qualitative assessment of the website would provide a more accurate picture in line with applicants’ experiences with these websites. The categories we chose, while attempting to be as comprehensive as possible, were based on those assessed in previous studies. It is still possible that more minor categories were missed that may be important to applicants. It is also important to consider that while these content categories offer a more objective way of viewing websites, plastic surgery residencies do not exist in a vacuum. Multiple factors play a role in delivery of information, and often involve not only information specific to a plastic surgery residency program but also information on the institution as a whole and its relationship to other services, such as general surgery.

Furthermore, while we collected data on social media use, we did not include it in our analysis, as the data required further evaluation. However, we recognize social media as an important force in the evolution of online content, and it is especially relevant in its potential role in recruiting applicants.^16^ This is also a cross-sectional study based solely on website presentation at the time that the search was conducted. We recognize that websites are not static, that most websites are conventionally updated annually, but that many programs are also limited by their technology departments in implementing changes. It is our hope that websites will move toward incorporating live content and updated data that is not only user-friendly but also easier for programs to update.

Overall, we found a discrepancy between the assessed websites and applicants’ self-reported needs, despite the importance of online resources^10,17^ to residency program applicants.^5,7,18,19^ “User-friendly” now represents more than availability of necessary information, but also encompasses the ability to engage, collaborate, and view information through various modalities. As the field of plastic surgery—and medicine as a whole—evolves, delivery of information and content must also evolve to fit ever-changing needs and behaviors,^13^ whether it be the use of social media or integration and automation of content. Our results suggest that even small changes with adding minor content such as unique program features can significantly improve applicants’ experiences with websites. Better website depiction of a program allows applicants to make more informed decisions on the most suitable program, which ultimately strengthens the quality of the residency match program. Further studies can qualitatively examine the aesthetic aspects and interactive interface of plastic surgery websites to better guide residency programs in designing their websites. They can also further explore the temporal relationship in the application process in progressing from away rotations to interviews to ranking, and the use of websites, especially in relation to other factors such as mentor suggestions, cost, or location. It is important to note that most applicants visit websites before the application process, which is approximately the same time as application to away rotations. In the end, quality residency program websites benefit both students and residency programs. For the students, websites hold potential for impacting students’ decision in choosing the right specialty and in choosing the right program. For residency programs, websites serve as vital communication tools in attracting quality applicants who best fit each individual program.

## Supplementary Material
