# Supplementary material for: Do Websites Provide What Applicants Need? Plastic Surgery Residency Program Websites Versus Applicant Self-reported Needs
Source: Plast Reconstr Surg Glob Open. 2018 Oct 2;6(10):e1900. doi: 10.1097/GOX.0000000000001900 (PMC6250462; doi:10.1097/GOX.0000000000001900)
Supplement: Supplementary file 1 [file gox-6-e1900-s001.pdf]

If you would like to be entered into a drawing for a $50 Amazon gift card, please provide us with a valid email address where the prize can be sent.

Please provide a valid email address

Type Email Address here

1/ Which social media apps do you use in your personal and daily life? *

Please select all social media apps that you use

- [ ] Facebook
- [ ] Twitter
- [ ] Instagram
- [ ] LinkedIn
- [ ] YouTube
- [ ] Google Plus
- [ ] Pinterest
- [ ] None

2/ How often do you use social media apps? *

please estimate how regularly you use social media apps

- [ ] Hourly
- [ ] Daily
- [ ] Weekly
- [ ] Every Two Weeks
- [ ] Monthly
- [ ] Annually

3/ Do/did you use residency program websites when researching residency programs? *

select one

- [ ] YES
- [ ] NO

THIS PAGE IS USING PREVIEW IMAGES FROM GETTY IMAGES. PLEASE PURCHASE THEM TO REMOVE THE WATERMARK.

please select all that apply

- [ ] No
- [ ] Brochures/print media
- [ ] Current/Previous Residents at the program of interest
- [ ] Current/Previous 4th year medical students at your home institution
- [ ] Medical School Advisors
- [ ] Another source: _______ (clarify below)

What source?

5/ Would you follow a residency program’s account on any of the following social media apps? *

select all social media apps you would follow

- [ ] Facebook
- [ ] Twitter
- [ ] Instagram
- [ ] LinkedIn
- [ ] YouTube
- [ ] Google Plus
- [ ] Pinterest
- [ ] I don't plan on following a residency program's social media

6/ Which social media app would you consider most useful in learning about a residency program? *

select one answer

- [ ] Facebook
- [ ] Twitter
- [ ] Instagram
- [ ] LinkedIn
- [ ] YouTube
- [ ] Google Plus
- [ ] Pinterest
- [ ] I don't believe social media is that useful to learning about a program

7/ When did you start browsing residency program

THIS PAGE IS USING PREVIEW IMAGES FROM GETTY IMAGES. PLEASE PURCHASE THEM TO REMOVE THE WATERMARK.

- [ ] Before medical school
- [ ] In selecting and considering specialties
- [ ] After deciding on a specialty but before the application process
- [ ] Only during the residency application process

8/ How useful was the information presented on residency program websites in your application process? *

select the response that applies most to you

- [ ] very useful
- [ ] moderately useful
- [ ] slightly useful
- [ ] not useful

9/ Did the quality of a residency program website influence your decision to apply to that particular program? *

select yes or no

- [ ] YES
- [ ] NO

10/ Did the quality of a residency program website influence your decision to interview at that particular program? *

select yes or no

- [ ] YES
- [ ] NO

11/ When viewing a program's web site, the following is important: *

Please select the option that applies most to you

Programmatic Content

Strongly Disagree	Disagree	Neutral	Agree	Strongly Agree
○	○	○	○	○

Quality of Photographs

THIS PAGE IS USING PREVIEW IMAGES FROM GETTY IMAGES. PLEASE PURCHASE THEM TO REMOVE THE WATERMARK.

Presence of Video Content

Strongly Disagree | Disagree | Neutral | Agree | Strongly Agree

Consistent Page Layout

Strongly Disagree | Disagree | Neutral | Agree | Strongly Agree

Aesthetic quality

Strongly Disagree | Disagree | Neutral | Agree | Strongly Agree

Ease of Navigation

Strongly Disagree | Disagree | Neutral | Agree | Strongly Agree

12/ When viewing a program's website, which information do you find most important *

please select all content entities that you find important

- [ ] Alumni testimonials and contact information
- [ ] Average resident work hours
- [ ] Board pass rates
- [ ] Career/Fellowship placement
- [ ] City/location information
- [ ] Current resident information
- [ ] Detailed application and interview information
- [ ] Faculty profiles
- [ ] FREIDA-like information
- [ ] Frequently asked questions and answers
- [ ] History of the program
- [ ] Hospital size and patient demographics

THIS PAGE IS USING PREVIEW IMAGES FROM GETTY IMAGES. PLEASE PURCHASE THEM TO REMOVE THE WATERMARK.

- [ ] Program goals and philosophy
- [ ] Residency curriculum
- [ ] Residency electives
- [ ] Resident finances/debt management
- [ ] Resident research
- [ ] Resident social lives
- [ ] Unique program features
- [ ] Work Schedules
- [ ]

13/ Was there any information not found on residency program websites that you would like to see? *

Please type your response, or No if nothing to add

Type Response Here

14/ Was there a discrepancy between any residency program's website and your actual interview experience/encounter with the respective program? *

please select yes or no

- [ ] YES
- [ ] NO

15/ What if any was your favorite or most memorable residency program website? *

please provide us with the program's name, or None for no response

Type Response Here

SUBMIT RESPONSES

THIS PAGE IS USING PREVIEW IMAGES FROM GETTY IMAGES. PLEASE PURCHASE THEM TO REMOVE THE WATERMARK.
